# Development of a Flow Injection Based High Frequency Dual Channel Quartz Crystal Microbalance

**DOI:** 10.3390/s17051136

**Published:** 2017-05-16

**Authors:** Jinxing Liang, Jing Zhang, Wenxiang Zhou, Toshitsugu Ueda

**Affiliations:** 1 Key Laboratory of Micro-Inertial Instrument and Advanced Navigation Technology, Ministry of Education, School of Instrument Science and Engineering, Southeast University, Nanjing 210096, China; 220142639@seu.edu.cn (J.Z.); 220162820@seu.edu.cn (W.Z.); 2 Research Center of the Graduate school of IPS, Waseda University, Kitakyushu 808-0135, Japan; t-ueda@waseda.jp

**Keywords:** dual-channel, QCM, high frequency, frequency interference, reference, flow cell

## Abstract

When the quartz crystal microbalance (QCM) is used in liquid for adsorption or desorption monitoring based bio- or chemical sensing applications, the frequency shift is not only determined by the surface mass change, but also by the change of liquid characteristics, such as density and viscosity, which are greatly affected by the liquid environmental temperature. A monolithic dual-channel QCM is designed and fabricated by arranging two QCM resonators on one single chip for cancelling the fluctuation induced by environmental factors. In actual applications, one QCM works as a specific sensor by modifying with functional membranes and the other acts as a reference, only measuring the liquid property. The dual-channel QCM is designed with an inverted-mesa structure, aiming to realize a high frequency miniaturized chip and suppress the frequency interference between the neighbored QCM resonators. The key problem of dual-channel QCMs is the interference between two channels, which is influenced by the distance of adjacent resonators. The diameter of the reference electrode has been designed into several values in order to find the optimal parameter. Experimental results demonstrated that the two QCMs could vibrate individually and the output frequency stability and drift can be greatly improved with the aid of the reference QCM.

## 1. Introduction

There has been a long history for quartz crystal microbalance (QCM) resonators to serve as a mass-frequency transducer, which vibrates in a thickness shear mode (TSM) mode. Own to its high sensitivity, stability, simple structure and low cost, QCMs has been widely applied in bio- or chemical sensing field [1,2,3,4]. Usually, the QCM resonator is composed of an ATcut quartz crystal piece and two metal film electrodes coated onto double surfaces of the quartz crystal chip. The fundamental resonance frequency of the QCM resonator is dominated by the crystal thickness and determined by Equation (1).

(1) $$f_{0}=\frac{\sqrt{\mu_{q}/\rho_{q}}}{2t}$$

where *t* is the quartz crystal thickness, $\mu_{q}$ and $\rho_{q}$ represent shear modulus of the AT-cut (2.947 × 10^11^ g·cm^−1^ s^2^) and the density of crystal (2.648 g·cm^−3^ for quartz), respectively.

When a rigid film is attached onto the QCM surface, which equivalently corresponds to an increase of crystal thickness, the fundamental frequency will drop accordingly. The resonance frequency shift is governed (in a limited film thickness) by Equation (2) derived by Sauerbrey [5]:

(2) $$\Delta f=-\frac{2f_{0}^{2}}{\sqrt{\mu_{q}\rho_{q}}}\cdot \frac{\Delta m}{A_{piezo}}$$

where Δf represents the frequency shift, $f_{0}$ is the fundamental resonance frequency, Δm is the attached mass, and $A_{piezo}$ is the piezoelectrically active area. Furthermore, when only one surface of the QCM is immersed in liquid, the resonance frequency is also affected by the density and viscosity of Newtonian liquid and the frequency shift is expressed as Equation (3) reported by Kanazawa et al. [6].

(3) $$\Delta f=-\frac{f_{0}^{3/2}}{\sqrt{\pi \mu_{q}\rho_{q}}}\cdot \sqrt{\eta_{L}\rho_{L}}$$

where $\eta_{L}$ and $\rho_{L}$ represent the liquid viscosity and density. The major advantage of QCM in sensing fields is the high frequency stability, besides the high sensitivity, simple structure, and low cost. However, temperature effect could not be neglected for high precise measurement [7]. When used in liquid, the quality factor (Q value) of QCM resonator will sharply drop due to the damping of liquid viscosity, which would reduce the frequency stability. Furthermore, the liquid mechanical properties such as density and viscosity are greatly affected by the liquid temperature. Obviously, the measurement environment fluctuation would cause large frequency drift leading to an imprecise measurement. Theoretically, the influence of environmental factors can be cancelled by arranging another QCM as a reference, which only monitors the environmental factors [8,9]. Actually, it is very difficult to find two identical QCMs having same resonating characteristics due to the different crystal blank, fabrication conditions and so on. Furthermore, separately arranged two QCM chip may receive a little different environment information, which causes non-precise compensation result. To avoid above mentioned problems, designing ofthe dual-channel QCM on one single quartz chip should be a suitable way which can also minimize the sensor chip size and saving the necessary sample volume [10]. In this case, the two QCM resonators can be expected to have the same resonance characteristics due to the same wafer and fabrication process. The environment caused frequency drift can be cancelled by taking the frequency difference between the two resonators. In actual applications, one channel of dual-QCM can be used as reference QCM which only outputs the frequency shift caused by liquid property, and finally it can be subtracted from the signals of the other working QCM. In the past decades, many researchers have studied and reported the designing and fabrication method or sensing applications on monolithic multichannel QCMs or QCM array [11,12,13,14]. In the case of monolithic dual-channel or multichannel QCM, the suppression of mechanical vibration coupling should be the most important issue, which is the prerequisite for the reference QCM to be as [15,16]. The frequency interference is mainly determined by the spacing between adjacent electrodes, the dimensions and thickness of each electrode, and geometry and frequencies of quartz resonators [17,18]. Several efforts can be made to reduce this kind of interference for modifying the multichannel sensor array configurations, such as mesa structure, inverted mesa structure, bi-mesa or bi-inverted-mesa structures [18,19].

On the other hand, the mass-frequency sensitivity of QCM is proportional to the square of the fundamental frequency, as shown in Equation (2). That means that higher mass-frequency sensitivity can be achieved by increasing the fundamental frequency. According to Equation (1), the fundamental frequency of QCM can be easily increased by reducing the corresponding crystal thickness, which is usually designed with an inverted-mesa structure. Previously, our group has reported the development of high frequency miniaturized QCM chip with an inverted-mesa structure fabricated by using a cheap wet etching process [20], and the application of flow injection based high frequency QCM system in bio sensing field [21].

In this research, we aim to develop a dual-channel high frequency QCM combined with the flow injection analysis technique. The frequency interference between the adjacent two QCM resonators is experimentally investigated by changing the electrode dimensions and the space between the two resonator electrodes. A novel spring pin based flow cell has been designed and fabricated for assembling the monolithic dual-channel QCM chip. The interference between two resonators is evaluated by combining the impedance analyzer and oscillator circuit. The compensation effect on output frequency drift is also tested in air and pure water by using two oscillator circuits.

## 2. Experimental

### 2.1. Design and Fabrication

**Figure 1:**
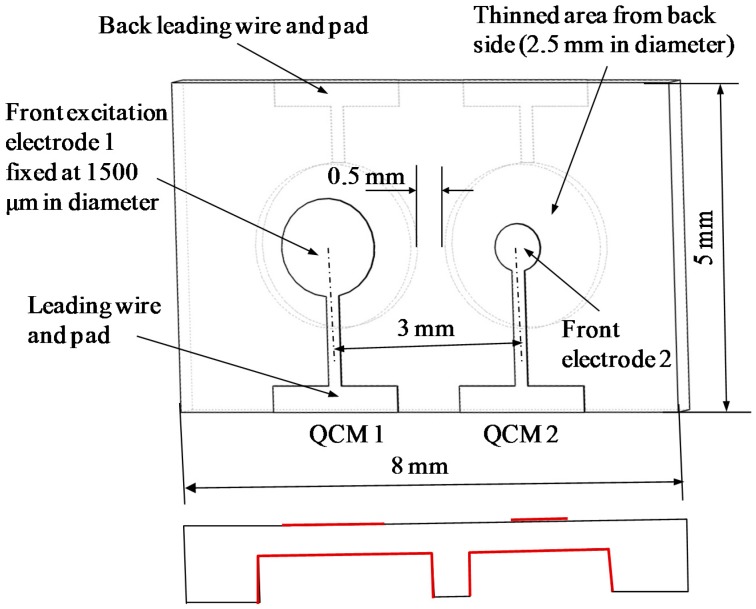
Schematic diagram of the dual-channel QCM configuration.

The monolithic dual-channel QCM on one chip is designed with an inverted-mesa structure as shown in Figure 1. Both the two high frequency QCM resonators vibrate only in the thinned area as previously reported. The two QCM resonators are arranged into separate thinned areas for avoiding too large thinned membrane, although it seems to save space by setting in one area. Furthermore, the existence of centering convex shape in wafer thickness between two QCM resonators can be expected to be benefit suppressing of frequency interference [14]. The vibrating area is thinned from the back side, and the front side keeps the original plane. The thinned area is fully coated with Au/Cr metal films as the backside excitation electrode for easy fabrication. That means all the electrodes on the front side are smaller than the electrodes on the etched back area.

The separate AT-cut quartz crystal chip used in this study is designed in rectangular shape with dimensions of 8 mm × 5 mm. The starting wafer is 40 mm × 40 mm in length and width, and 100 μm in thickness with polished double planar faces. The fundamental frequency of QCM resonator is about 35 MHz with a vibration area thickness around 48 μm. The excitation electrodes are composed of Au/Cr bi-layer metal films, a 100 nm thick gold film as the main electrode and a 20 nm thin chromium film as an adhesion layer between quartz and gold film. The thinned areas are designed to be 2500 μm in diameter and the space of the two thinned area is fixed to be 3000 μm, which means a minimum 0.5 mm wide convex wall exists between the two resonators. As for the designing of the front excitation electrodes on the dual channel chip, one electrode is fixed at 1500 μm in diameter and the other varies from 750 μm to 2400 μm at an interval of 150 μm. The varied excitation electrode dimension on one side means different spaces between the two excitation electrodes, which is considered to be one of the important parameters for reducing the frequency interference. Twenty four single dual-channel QCM chip can be cut out of one quartz blank. The thinning area is etched by using well established quartz wet etching process, using saturated bifluoride ammonium solution at 85 degrees Celsius [20].

### 2.2. Evaluation

**Figure 2:**
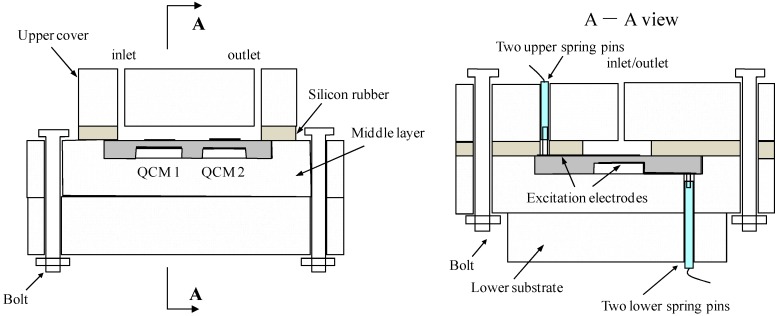
Schematic illustration flow cell for the dual-channel QCM sensor, the right one is the A-A view of the left one.

A micro flow cell is designed and fabricated to assemble the fabricated resonator chip, as shown in Figure 2. The flow cell consists of three parts: the upper cover, the middle layer and the lower substrate, which were made from polymethyl methacrylate (PMMA). A rectangular cavity 95 μm in depth is created in the middle layer used to stabilize and hold the QCM chip. A 1 mm thick silicon rubber, which is drilled through in the centering area exposing to the electrodes of the QCM resonators, is adhered onto the back side of the upper cover.

The designed flow cell configuration makes it easy and changeable to assemble the QCM chip. Firstly, the sensor chip is placed into the cavity of the middle layer. Secondly, the upper layer, equipped with two spring pins, is jointed with the middle layer by using two bolts, ensuring the spring pins contacting on the pads of the QCM excitation electrodes. Finally, the lower layer, also equipped with two spring pins is held together with the middle layer by using bolts, leading out the QCM electrodes on the back side. The liquid can be introduced into the cell through the inlet and outlet ports in the upper PMMA cover.

An impedance analyzer 4294A is used to measure vibration characteristics including Q value, equivalent circuit parameters and resonance frequency. A two channel high frequency oscillator is developed for driving the QCM resonators as introduced before [21]. A flow injection system is established, which is mainly composed of a syringe pump, a sample injector, flow cell and impedance analyzer or self-made oscillator circuit. In addition, all the measurement is carried out at room temperature.

## 3. Results and Discussion

**Figure 3:**
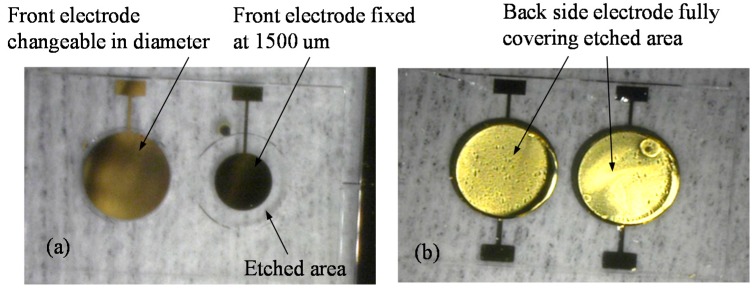
Example images of the fabricated dual-channel QCM chip: (**a**) Front view; (**b**) Back view.

Figure 3 shows an example picture of fabricated dual-channel QCM chip. On the front flat side, the electrode of channel 1 is fixed at 1500 μm, and the electrode of channel 2 is designed to be changeable. Figure 3b shows the back side electrodes, which fully cover the etched area.

Excitation electrodes can be electrically connected with impedance analyzer or oscillator circuit by contacting the spring pins on the pads. At first, the vibration characteristics of each channel resonator are measured separately by using impedance analyzer to ensure each resonator could resonate well individually.

Generally speaking, for a given wafer thickness (deciding the fundamental frequency) and vibration area, too small electrode suffers from bad energy trapping and too large electrode will easily induce spurious vibration, both wouldcause the droping of Q value. In this study, the etching area is designed to be 2500 μm in diameter, the measured results indicates that the electrode dimension larger than 2100 μm will cause a sharp drop of Q value (1386) due to the small distance between the electrode and the edge of vibration area as we discussed before [20]. Accordingly, the frequency stability becomes worse, and the standard deviation was measured to be large as 53 Hz, which is considered too large for actual application.

To investigate the interference between two channels, the most effective and direct way is to coat thin film on the one channel and observe the frequency shift of the other channel. However, for such a small dual channel chip, a special tool is needed to separately treat the two resonators. In this research, the vibration coupling of two channels is evaluated by comparing frequency shift of one channel before and after exciting the resonation of the other channel, as introduced in [22]. The detailed experimental procedure is described as follows: (1) Channel 1 (1500 μm electrode) of dual channel QCM (DQCM) is connected with impedance analyzer and channel 2 is kept free, and the resonance frequency of channel 1 is recorded by using a Labview-based data acquisition software; (2) After being stabilized for at least 20 min, the channel 2 (changeable electrode) is excited by using the tailor-made oscillator circuit, and the resonance frequency of channel 1, measured by impedance analyzer is continually recorded. Each measurement lasts about one hour. The data measured by impedance analyzer can be used to estimate the stability of sensor’s fundamental frequency. By analyzing the data before and after the channel 2 is connected with oscillator circuit, the influence of channel 2 on the vibration of channel 1 can be concluded. In the same way, the influence of channel 1 on the vibration of channel 2 is also measured.

Three typical dual-channel chips, the channel 2 electrodes of which are 1050 μm (chip 1), 1500 μm (chip 2) and 1950 μm (chip 3), respectively, are selected to measure the interference between two resonators. Each resonator has been confirmed to own a Q value larger than 10,000. The measured conductance spectrums of three pairs of 35 MHz dual-channel QCMs are summarized in Table 1. Herein, *f*_0_ represents the fundamental resonance frequency at maximum conductance, and G represents the conductance. And the measurement is taken individually for the two channels, which demonstrated that the dual-channel QCMs could vibrate individually when assembled into flow cell in air.

**Figure 4:**
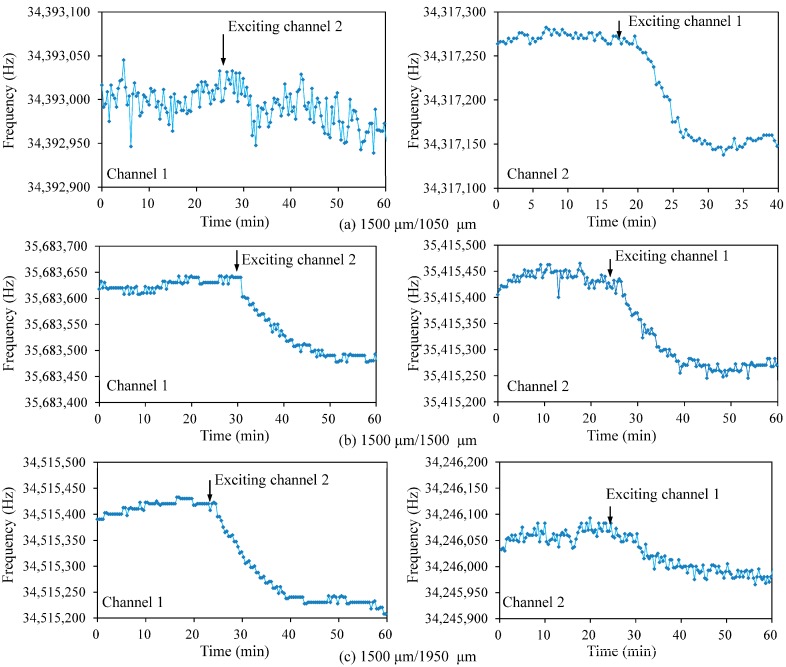
Measured frequency interference between two channels.

Figure 4 shows the measured interference results. The standard deviation values are calculated by using the data during stable oscillation (20 min), which are recorded by impedance analyzer before and after exciting the other resonator. The Δ*f* is the frequency difference for the same channel before and after exciting the other channel (see Table 1).

The largest frequency shift appears on the chip 2, the two channels of which have the same electrode dimension. The frequency interferences of the other two pairs (chips 1 and 3) with different electrodes are smaller than the chip 2, which agrees well with the previous report [15]. Furthermore the chip 1 shows smaller interference than chip 3, which is considered that chip 1 has larger distance than chip 3due to its small electrode 2.

The small interference should thank to the inverted-mesa structure. Firstly, the thinned vibration area (high frequency) gives a relatively high distance to thickness ratio, which is wanted for reducing interference [15]. Secondly, the convex-wall between the two resonators with inverted-mesa structures will contribute to the suppression of interference [23].

It can be learnt from Table 1 and Figure 4 that the frequency shift caused by interference for the two resonators on one chip is different, and the resonator with smaller electrode is affected more. This could be interpreted that the resonator with smaller electrode havs smaller vibration inertia, so it is easier to be affected by the neighbored large one. According to these results, the chip 1 (1500 μm/1050 μm) is preferred for sensor applications. In this case, QCM 1 with large electrode (1500 μm) acts as reference and QCM 2 with small electrode (1050 μm) works as sensing element. The interference from the sensing element is less than 0.76 ppm, which is considered a negligible level.

The reference effect on reducing frequency drift of dual-channel QCMs has been tested in both air and deionized pure water by taking the differential value of two channels. The sensor chip with the same diameter of electrode (1500 μm) was used, and both channels were driven by using separate oscillator circuits. All the experiments were carried out at room temperature.

**Figure 5:**
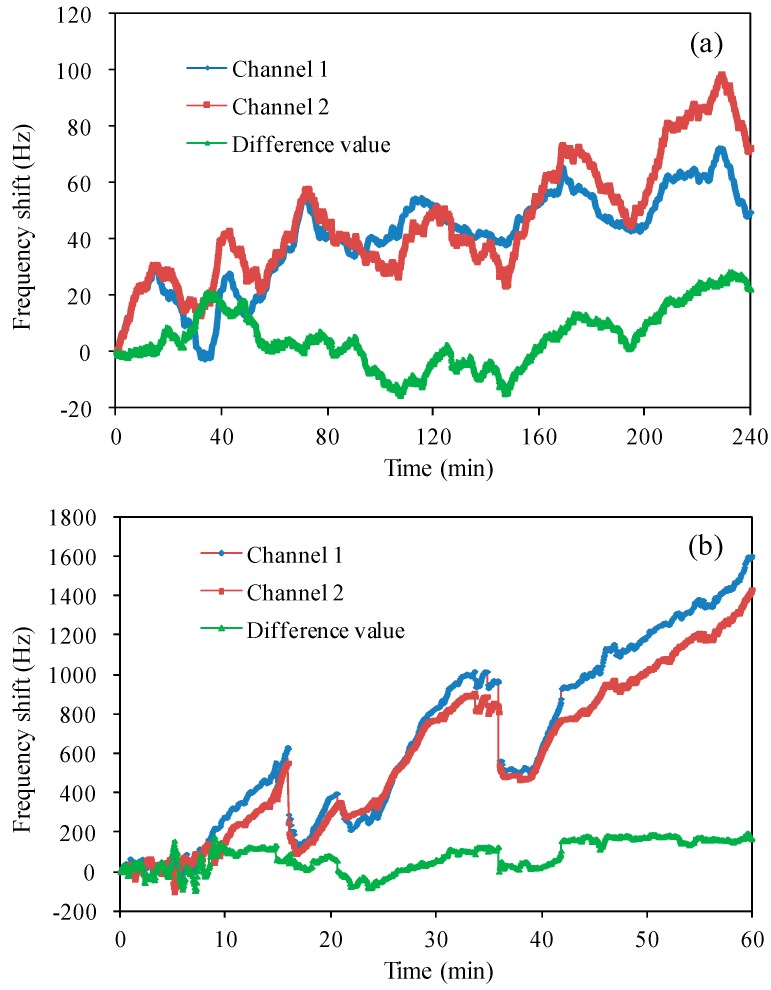
Measured frequency shift with oscillator circuit: (**a**) in air; (**b**) in pure water.

Figure 5a shows the oscillation frequencies of two QCM resonators and the frequency difference in air for 4 h. Both the two resonance frequencies drifted about 100 Hz in the same trend, which is considered to be caused by the environment factors, especially temperature fluctuation. By taking the frequency difference, the drift can be observed to be about 20 Hz. Figure 5b demonstrates the frequency drift of two channels in liquid for 1 h. About 1500 Hz was observed for each channel, which means more complex environment condition in liquid, such as the affection of liquid density and viscosity. By taking the frequency difference, the drift was reduced to be below 200 Hz.

These two experimental results demonstrated the good reference effect of dual-channel QCM even using the largest interference chip 2. This result should thank the monolithic configuration, which guarantees the same resonance characteristics of the two resonators. Furthermore, the frequency stability was also compared by taking the frequency average value, standard deviation for individual QCM resonator and the frequency differences. These measured results are summarized in Table 2.

It can be clearly concluded that the frequency stability was also greatly improved by taking the difference value. However, the measured noise of each channel is still larger than the reported QCM sensors, which is mainly caused by our developing high frequency oscillator circuit, and the connection method between the flow cell and circuit board. Aiming to actual applications, we are currently focusing on reducing the output noise by improving oscillator circuit and data acquisition method.

## 4. Conclusions

The designing and fabrication of high frequency dual-channel QCM was reported. The inverted-mesa structure with a middle convex wall was used to suppress the frequency interference. The full QCM chip is designed to be with a small size below 5 × 8 mm^2^ resonating at 35 MHz. The effect of electrode size on the resonator characteristics and the interference were experimentally investigated. A flow injection system has been established for demonstrating actual applications. For the 35 MHz resonators with an etching area of 2.5 mm in diameter, dual-channel QCM with 1500 μm/1050 μm electrodes showed the lowest interference, which is less than 0.76 ppm. Imitating the actual application condition in air and liquid, the frequency stability and drift of fabricated dual-channel QCM were measured by using tailor-made oscillator circuit. The greatly improved frequency difference value strongly suggested that one chip dual channel QCM has the potential to be used for bio- or chemical sensor.

## Figures and Tables

**Table 1:** Measured characteristics of Dual-channel QCM.

Parameter	Electrode Diameter (μm)					
	1500	1050	1500	1500	1500	1950
*f*_0_ (MHz)	34.40	34.31	35.68	35.42	34.52	34.22
Q factor	10,820	31,492	12,644	17,013	14,253	10,782
G (ms)	17.96	28.22	20.50	28.89	24.07	23.74
Standard deviation (before connecting oscillator) (Hz)	16	5	10	14	12	13
Standard deviation (after connecting oscillator) (Hz)	21	27	15	9	20	16
Δ*f* (Hz)	26	102	141	168	169	64

**Table 2:** Frequency shift of dual-channel QCMs in air and deionized water.

		Channel 1 (Hz)	Channel 2 (Hz)	Difference Value (Hz)
Air	Average value	42.14	48.44	6.30
	Standard deviation	16.00	20.48	10.64
Deionized water	Average value	671.86	588.77	83.09
	Standard deviation	455.23	399.47	74.57
